# Effects of ranolazine on right ventricular function, fluid dynamics, and metabolism in patients with precapillary pulmonary hypertension: insights from a longitudinal, randomized, double-blinded, placebo controlled, multicenter study

**DOI:** 10.3389/fcvm.2023.1118796

**Published:** 2023-06-13

**Authors:** Q. Joyce Han, Paul Forfia, Anjali Vaidya, Gautam Ramani, Robert A. deKemp, Robert H. Mach, David A. Mankoff, Paco E. Bravo, Marcelo DiCarli, Stephen Y. Chan, Aaron B. Waxman, Yuchi Han

**Affiliations:** ^1^ Cardiovascular Division, Massachusetts General Hospital, Boston, MA, United States; ^2^ Pulmonary Hypertension, Right Heart Failure, and CTEPH Program, Department of Cardiology, Temple University Hospital, Philadelphia, PA, United States; ^3^ Cardiovascular Division, University of Maryland, Baltimore, MD, United States; ^4^ Cardiac PET Center, University of Ottawa Heart Institute, Ottawa, ON, Canada; ^5^ Department of Radiology, University of Pennsylvania, Philadelphia, PA, United States; ^6^ Cardiovascular Division, University of Pennsylvania, Philadelphia, PA, United States; ^7^ Cardiovascular Division, Brigham and Women’s Hospital, Boston, MA, United States; ^8^ Center for Pulmonary Vascular Biology and Medicine, Pittsburgh Heart, Lung, Blood, and Vascular Medicine Institute, Division of Cardiology, Department of Medicine, University of Pittsburgh Medical Center and University of Pittsburgh School of Medicine, Pittsburgh, PA, United States; ^9^ Center for Pulmonary Heart Disease, Pulmonary and Critical Care Medicine, Brigham and Women’s Hospital, Boston, MA, United States; ^10^ Cardiovascular Division, Wexner Medical Center, The Ohio State University, Columbus, OH, United States

**Keywords:** pulmonary hypertension, cardiac magnetic resonance imaging, 4D flow MRI, metabolism, positron emission tomography

## Abstract

**Introduction:**

Right ventricular (RV) function is a major determinant of outcome in patients with precapillary pulmonary hypertension (PH). We studied the effect of ranolazine on RV function over 6 months using multi-modality imaging and biochemical markers in patients with precapillary PH (groups I, III, and IV) and RV dysfunction [CMR imaging ejection fraction (EF) < 45%] in a longitudinal, randomized, double-blinded, placebo-controlled, multicenter study of ranolazine treatment.

**Methods:**

Enrolled patients were assessed using cardiac magnetic resonance (CMR) imaging, ^11^C-acetate and ^18^-F-FDG positron emission tomography (PET), and plasma metabolomic profiling, at baseline and at the end of treatment.

**Results:**

Twenty-two patients were enrolled, and 15 patients completed all follow-up studies with 9 in the ranolazine arm and 6 in the placebo arm. RVEF and RV/Left ventricle (LV) mean glucose uptake were significantly improved after 6 months of treatment in the ranolazine arm. Metabolomic changes in aromatic amino acid metabolism, redox homeostasis, and bile acid metabolism were observed after ranolazine treatment, and several changes significantly correlated with changes in PET and CMR-derived fluid dynamic measurements.

**Discussion:**

Ranolazine may improve RV function by altering RV metabolism in patients with precapillary PH. Larger studies are needed to confirm the beneficial effects of ranolazine.

## Introduction

Right ventricular (RV) function is a major determinant for the outcome in patients with pulmonary arterial hypertension (PAH). In the setting of increased afterload, the RV may initially compensate by hypertrophy to maintain cardiac output; as disease progresses, the RV decompensates and undergoes dilatation, which eventually lead to RV failure and death. Many neurohormonal changes occur in the process, but little is known about the connection between hemodynamic changes and molecular signaling pathways. Particularly, the irreversible molecular changes associated with chronic excess afterload. Current therapeutic interventions in PAH are focused on the pulmonary vasculature; yet, survival is significantly associated with RV function at presentation and its response to therapy (1). Therefore, the understanding of cellular and molecular signals responsible for RV decompensation is of particular interest.

The clinical assessment of the RV is currently based on volumetric measurements from CMR (such as RVEF, RV mass, etc.), and emerging new imaging techniques provide insights into additional pathophysiology, such as fluid dynamics and metabolism. Three-directional velocity-encoded phase contrast MRI (here termed 4D flow MRI) is a non-invasive technique that provides quantitative measurements of the whole heart velocity field over time. Using 4D flow MRI, we previously demonstrated that, compared to healthy adults, PAH patients not only had RVs that generated lower kinetic energy during systole, but also had significantly higher energy loss in the form of viscous dissipation in the pulmonary arteries (2). Positron emission tomography (PET) using 2-deoxy-2-[^18^F] fluoro-D-glucose (FDG) is applied to measure glucose uptake as an indicator of glucose metabolism in the right heart and pulmonary vasculature (3). In a prospective clinical study, RV FDG uptake correlated with RV fractional area changes measured by echocardiography in PAH patients in one-year follow-up period (4).

Ranolazine is a second-line agent in the management of stable angina without negative chronotropic, dromotropic, or inotropic activity at rest or exercise. Its full mechanism of action as an anti-anginal agent is not completely understood. It inhibits the late phase inward sodium current (I_Na_) in cardiomyocytes (the specific subunit is almost exclusively expressed in the myocardium), decreasing intracellular calcium, and subsequently improving coronary blood flow (5). Additionally, ranolazine may improve myocardial ischemia by altering cellular metabolism. It has been suggested that ranolazine may partially inhibit fatty acid oxidation and activates pyruvate dehydrogenase, thereby shifting the balance to glucose oxidation, consuming less oxygen (6–8). Ranolazine is not approved for clinical use in patients with PAH, but open-label studies have shown its safety and potential efficacy in symptomatic and functional improvement at 3 months (9, 10). We recently showed significant improvement in RVEF in PAH patients treated with ranolazine for 6 months (11).

In this report, we describe the effects of ranolazine treatment over 6 months on (1) the RV and PA fluid dynamic profile, (2) ventricular glucose uptake and oxygen consumption, and (3) metabolomic profile measured in peripheral blood, in patients with precapillary PH.

## Materials and methods

We enrolled patients with precapillary PH and stable background therapy with persistent RV dysfunction [CMR imaging ejection fraction (EF) of <45%], in a longitudinal, randomized, double-blinded, placebo controlled, multicenter study of ranolazine treatment from 2014 to 2017. Detailed study design, inclusion/exclusion criteria, patient demographics, and data acquisition were previously published (12).

### Fluid dynamic analysis

RV kinetic work density and PA energy loss were introduced in our prior work (2). In brief, RV work density is established as the minimum amount of kinetic work required per stroke volume (SV) in a single cardiac cycle, and PA energy loss (E_loss_PA_) measures the irreversible loss of mechanical energy in the PA in the form of viscous dissipation. The percentage of energy loss in the total KE output can thus be calculated by the ratio of totally dissipated energy and total KE output: E_loss_PA_/KE_out_.

### PET data acquisition and analysis

PET was used to quantify regional myocardial blood flow (MBF) and oxygen consumption at rest using ^11^C-acetate and, myocardial glucose uptake, using ^18^F-FDG. Imaging was performed using a whole-body PET-CT scanner (Gemini TF or Gemini TF BigBore [Philips Medical Systems, Netherlands] or Siemens mCT [Siemens Healthineers, Germany]). Each subject in the study fasted overnight for at least 12 h and abstained from coffee, tea, and tobacco for at least 12 h.

Myocardial oxidative metabolism and blood flow were measured with ^11^C-acetate (20–25 mCi), given as an intravenous bolus with a simultaneous start of a 30-min dynamic acquisition of PET list mode images. The rate of ^11^C-acetate uptake (K_1_) was measured using FlowQuant software (Ottawa Heart Institute, Canada) and absolute quantification of regional MBF (in ml/min/g) was performed using the dynamic data acquired between 0 and 5 min following ^11^C-acetate injection according to the method of van den Hoff et al. (13) Intracellular ^11^C-acetate is rapidly converted to ^11^C-acetyl-CoA in the mitochondria where it enters the tricarboxylic acid cycle and is oxidized with the radiolabel washing out as ^11^C-CO_2_. The rate constant of a mono-exponential clearance function (K_MONO_) was fit to the ^11^C-clearance curve using FlowQuant software (Ottawa Heart Institute, Canada) and used to calculate myocardial oxygen consumption (MVO_2_) using the method of Sun et al. (14).

In order to standardize myocardial glucose utilization, an oral glucose load with 50 grams of Glucoa was performed 60 min before ^18^F-FDG injection. Fingerstick glucose was obtained to ensure glucose level was ≤125 mg/dl for ^18^F-FDG (10 mCi) administration. Patients rested for 60 min before the static FDG images were acquired for 15 min according to the guidelines of the Society of Nuclear Medicine and Molecular Imaging (SNMMI), American Society of Nuclear Cardiology (ASNC), and Society of Cardiovascular Computed Tomography (15); mean and maximum of the standardized uptake value (SUV) for the whole myocardium were calculated as described in the guideline.

### Metabolomics

Plasma samples were collected at baseline and after 26 weeks from all study subjects, and global biochemical profiles were determined using Metabolon Inc (Morrisville, NC, USA). The dataset comprises 1,006 compounds of named biochemicals. Log transformation and imputation of missing values, if any, with the minimum observed value for each compound were performed. A two-way ANOVA with repeated measures was used to identify biochemicals that differed significantly between experimental groups. Pathway analyses of the numbers of biochemicals that achieved statistical significance (*p *≤ 0.05) were given. An estimate of the false discovery rate (*q*-value) is calculated to consider the multiple comparisons that normally occur in metabolomic-based studies.

### Statistics

Collected data were summarized using descriptive statistics. For continuous variables, mean, standard deviation (SD), minimum and maximum values were presented. Scatter plots were used to visually display the relationships between variables of interest. The relationship between CMR, PET, metabolomics measurements were assessed using Pearson correlation analyses. Individual compounds with a false discovery rate of <10% in metabolomics and key related significant compounds in pathway analyses were correlated with CMR and PET measurements.

## Results

### Patient population

**Figure 1:**
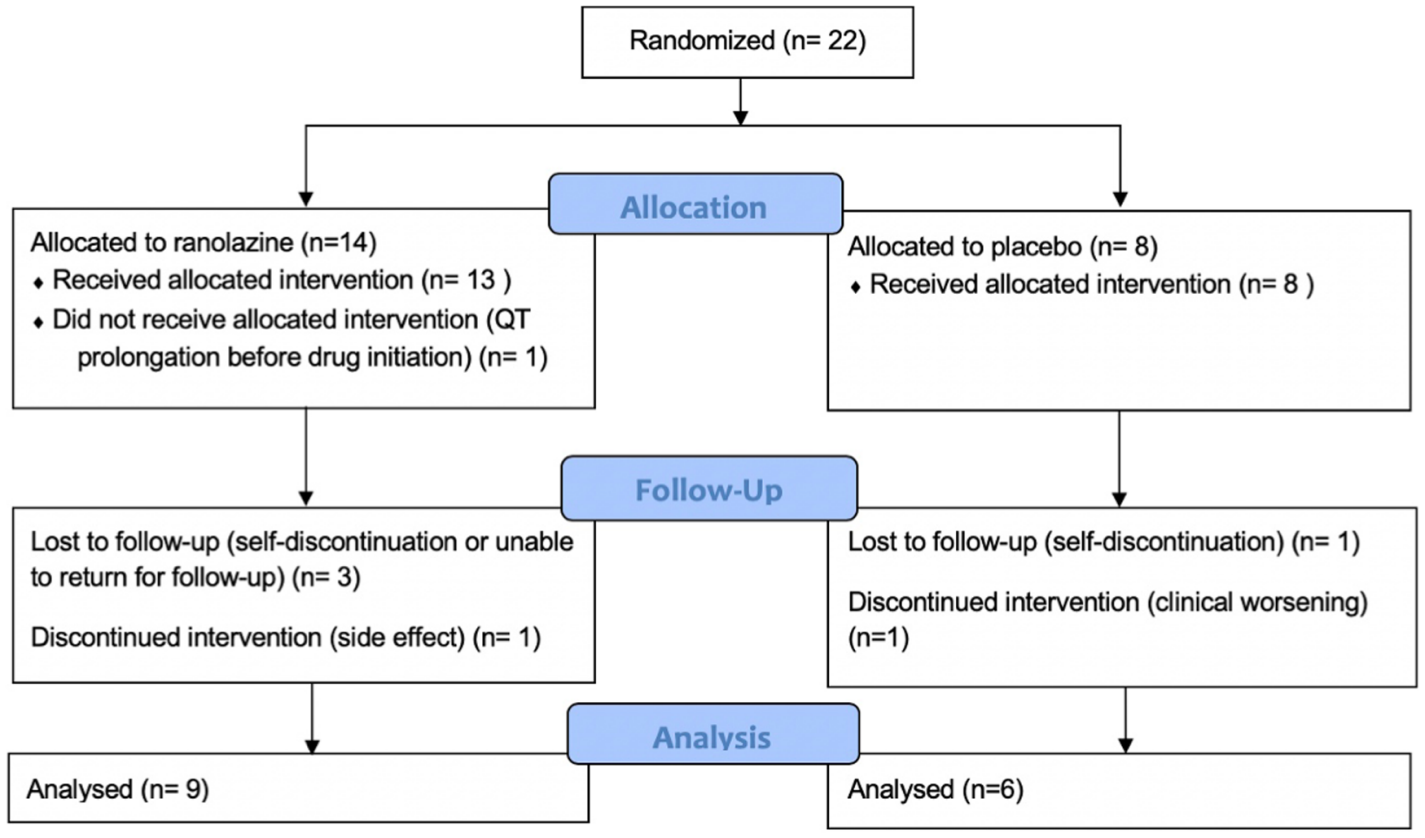
Study enrollment and follow up information. Seven patients did not complete follow-up study owing to QT prolongation before initiation of therapy (*n* = 1), self discontinuation (*n* = 2), unable to return for follow-up visit (*n* = 2), clinical worsening (*n* = 1), and side effects (*n* = 1). There is no statistical difference between the discontinuations in the two groups (*p* = 0.17).

The details of the study population were previously published (12). We performed CMR screening in 38 patients and enrolled 22 patients. Screen failures were due to normal RVEF (*n *= 14) and QT prolongation (*n *= 2). Baseline characteristics including all enrolled patients are presented in Table 1. As noted in Figure 1, 14 patients were randomized to ranolazine and 8 was randomized to placebo with an intention to randomize 2:1. Seven patients did not complete follow-up imaging studies due to QT prolongation before initiations of therapy but after randomization (*n* = 1, ranolazine group), self-discontinuation (*n* = 2, one in each group), unable to return for follow-up visit (*n* = 2, ranolazine group), discontinuation due to side effect (*n* = 1, ranolazine), discontinued due to clinical worsening (*n* = 1, placebo). There was no statistical difference in the drop-out in each of the categories in the two groups (*p* = 0.17).

**Table 1:** Summary of the study population.

	Placebo (*n *= 8)	Ranolazine (*n *= 14)	*P* value
**Demographics**			
Age (years)	53.6 ± 14.07 (22.0–68.0)	55.4 ± 16.93 (24.0–78.0)	0.809
Female sex	5 (62.5%)	8 (57.1)	>.999
White race	6 (75.0)	9 (64.3)	>.999
6-Minute walk distance (m)	385.6 ± 179.8 (85.3–547.0)	302.3 ± 113.7 (140.2–477.0)	0.1954
NT-proBNP (pg/ml)	1,644.6 ± 928.9 (202.0–2,979.0)	946.7 ± 933.7 (51.0–2,620.0)	0.1066
Creatinine (mg/dl)	0.91 ± 0.36 (0.20–1.49)	1.09 ± 0.35 (0.52–1.71)	0.2698
**Clinical diagnosis**			
CTEPH	0	2 (14.3)	
Congenital heart disease related PH	1 (12.5)	0	
Connective tissue disease related PH	1 (12.5)	2 (14.3)	
Hereditary PAH	1 (12.5)	0	
Idiopathic PAH	5 (62.5)	9 (64.3)	
PH with COPD	0	1 (7.1)	0.627
**NYHA functional class**			
II	5 (62.5)	6 (42.9)	
III	2 (25)	6 (42.9)	
IV	1 (12.5)	2 (14.3)	0.8266
**PAH medication**			
Prostanoid	6 (75)	7 (50)	0.3802
Phosphodiesterase type 5 inhibitor	6 (75)	13 (92.9)	0.2545
sGC stimulator (Riociguat)	1 (12.5)	1 (7.14)	0.4848
Endothelin receptor antagonist	7 (87.5)	8 (57.1)	0.1932

CTEPH, chronic thromboembolic pulmonary hypertension; COPD, chronic obstructive pulmonary disease; PAH, pulmonary arterial hypertension; NYHA, New York Heart Association; sGC, soluble guanylate cyclase.

### CMR and PET

**Figure 2:**
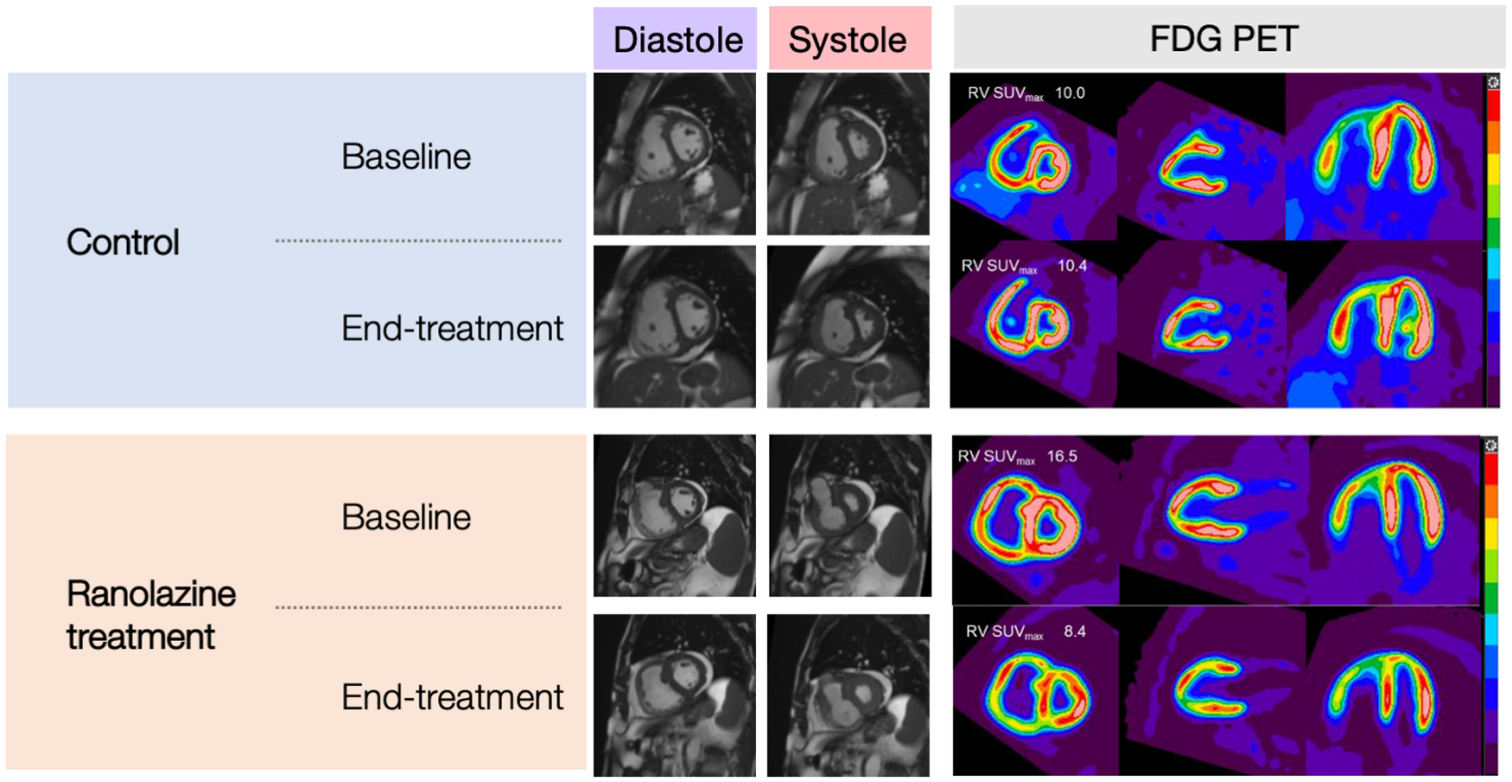
CMR images (SAX, mid-ventricular level) and C-11 PET images (SAX, 2-chamber, and 4-chamber) of subjects in the control group and ranolazine treatment group.

Results for CMR and PET are summarized in Table 2 and Figure 2. Changes in RVEF, RVSV, and LVSV in ranolazine treated patients was significantly greater compared to those in placebo treated patients (*p = 0.005*) as previously reported and discussed (12). There was no significant difference in time-dependent changes in RV kinetic energy profile or PA energy loss between the treatment and the placebo arms. Similarly, no statistically significant time-dependent differences were noted in the MBF, K_mono_, MVO_2_, SUV mean, or SUV max between the treatment and the placebo arms. The 6-month change in RV/LV SUV mean ratio in the ranolazine group was borderline significantly greater compared to that in the placebo group (−0.17 ± 0.14 vs. 0.01 ± 0.12, *p* = 0.053).

**Table 2:** Summary of measurements obtained by CMR and PET in placebo compared to ranolazine.

	Placebo			Ranolazine			*P* Value
	Baseline Mean (SD)	End-treatment Mean (SD)	Change from baseline (SE)	Baseline Mean (SD)	End-treatment Mean (SD)	Change from baseline (SE)	
**RV**							
EF (%)	23.4 (9.8)	22.5 (8.2)	−4.0 (2.2)	34.1 (4.9)	39.6 (5.1)	7.6 (1.7)	**0**.**005**
EDV (ml)	343.51 (73.0)	327.3 (68.2)	14.3 (19.0)	244. 9 (46.2)	246.2 (42.8)	−19.1 (14.2)	0.266
EDVi (ml /m^2^)	186.9 (38.3)	171.6 (53.6)	−21.3 (15.1)	125.7 (33.1)	129.9 (36.5)	8.2 (11.1)	0.226
ESV (ml)	266.9 (76.3)	257.8 (77.5)	17.8 (19.8)	162.5 (38.9)	149.2 (31.1	−31.2 (14.7)	0.128
ESVi (ml /m^2^)	145.9 (43. 7)	135.5 (53.8)	−10.0 (13.8)	82.7 (21.8)	78.3 (21.9)	−4.8 (10.2)	0.806
SV (ml)	76.6 (19.7)	69.4 (13.8)	−8.3 (4.8)	82.4 (13.4)	97.0 (17.7)	15.3 (3.9)	**0**.**004**
RV KE work density (mJ/ml)	55.3 (45.6)	43.8 (32.09)	20.8 (20.7)	6.6 (42.9)	45.7 (34.5)	20.5 (28.1)	0.64
PA energy loss (mW)	9.2 (6.0)	11.0 (6.8)	0.8 (2.9)	7.7 (3/4)	6.7 (2.5)	−1.0 (0.96)	0.46
Flow (ml/min/g)	1.5 (0.6)	2.3 (2.8)	0.77 (2.5)	1.2 (0.47)	1.2 (0.4)	0.09 (0.6)	0.95
MVO2 (Kmono) (1/min)	0.06 (0.01)	0.06 (0.02)	0.02 (0.02)	0.06 (0.009)	0.06 (0.01)	0.10 (0.03)	0.39
MVO2 (ml/min/g)	11.6 (2.3)	13.8 (3.2)	3.4 (4.7)	11.9 (1.8)	11.4 (2.8)	1.9 (5.0)	0.39
SUVmean	6.6 (3.5)	5.9 (2.6)	−1.6 (3.2)	6.1 (2.9)	4.5 (3.0)	−2.5 (3.9)	0.95
SUVmax	10.1 (6.1)	9.3 (4.6)	−2.3 (5.4)	10.02 (5.6)	7.5 (4.9)	−4.3 (7.9)	0.84
**LV**							
EF (%)	54.6 (2.9)	55.8 (6.0)	0.9 (1.4)	52.7 (10.1)	56.7 (10.2)	4.3 (1.1)	0.095
EDV (ml)	136.4 (22.9)	131.4 (7.3)	−6.5 (8.7)	157.7 (41.0)	172.4 (48.8)	15.8 (7.0)	0.081
EDVi (ml /m^2^)	73.4 (5.2)	69.3 (16.6)	−5.0 (4.4)	79.4 (17.5)	88.9 (21.3)	10.1 (3.5)	0.025
ESV (ml)	61.8 (10.3)	57.8 (5.9)	−4.2 (5.1)	77.9 (38.4)	77.7 (41.8)	−0.02 (4.1)	0.544
ESVi (ml /m^2^)	33.3 (1.4)	30.6 (8.9)	−3.0 (2.7	38.1 (13.5)	38.9 (14.8)	1.0 (2.2)	0.276
SV (ml)	74.6 (14.2)	73.5 (11.6)	−2.7 (4.1)	79.8 (10.6)	94.4 (12.9)	15.6 (3.4)	**0**.**007**
Flow (ml/min/g)	1.6 (0.5)	1.7 (0.6)	0.19 (0.8)	1.4 (0.5)	1.5 (0.4)	0.20 (0.3)	0.84
MVO2 (Kmono) (1/min)	0.07 (0.01)	0.07 (0.02)	0.02 (0.03)	0.07 (0.01)	0.07 (0.02)	0.01 (0.03)	0.55
MVO2 (ml/min/g)	13.1 (2.4)	15.1 (3.1)	3.5 (5.3)	14.2 (2.2)	13.1 (3.1)	2.2 (6.3)	0.55
SUVmean	9.5 (4.2)	8.3 (2.5)	−0.23 (4.5)	8.7 (3.0)	7.9 (5.1)	−1.5 (5.8)	0.64
SUVmax	14.6 (7.3)	12.6 (4.2)	−3.6 (7.9)	14.1 (5.8)	12.2 (8.2)	−3.7 (11.3)	0.95
RV/LV SUVmean ratio	0.68 (0.19)	0.69 (0.18)	0.007 (0.12)	0.67 (0.20)	0.56 (0.15)	−0.17 (0.14)	**0**.**05**
RV/LV SUVmax ratio	0.68 (0.21)	0.71 (0.24)	0.02 (0.14)	0.68 (0.22)	0.59 (0.17)	−0.12 (0.19)	0.46

LVEDV, left ventricular end-diastolic volume; LVEDVi, left ventricular end-diastolic volume index; LVEF, left ventricular ejection fraction; LVESV, left ventricular end-systolic volume; LVESVi, left ventricular end-systolic volume index; RVEDV, right ventricular end-diastolic volume; RVEDVi, right ventricular end-diastolic volume index; RVEF, right ventricular ejection fraction; RVESV, right ventricular end-systolic volume; RVESVi, right ventricular end-systolic volume index; SUV, standard uptake value.

Values listed in bold indicate statistical significance.

### Metabolomics

**Figure 3:**
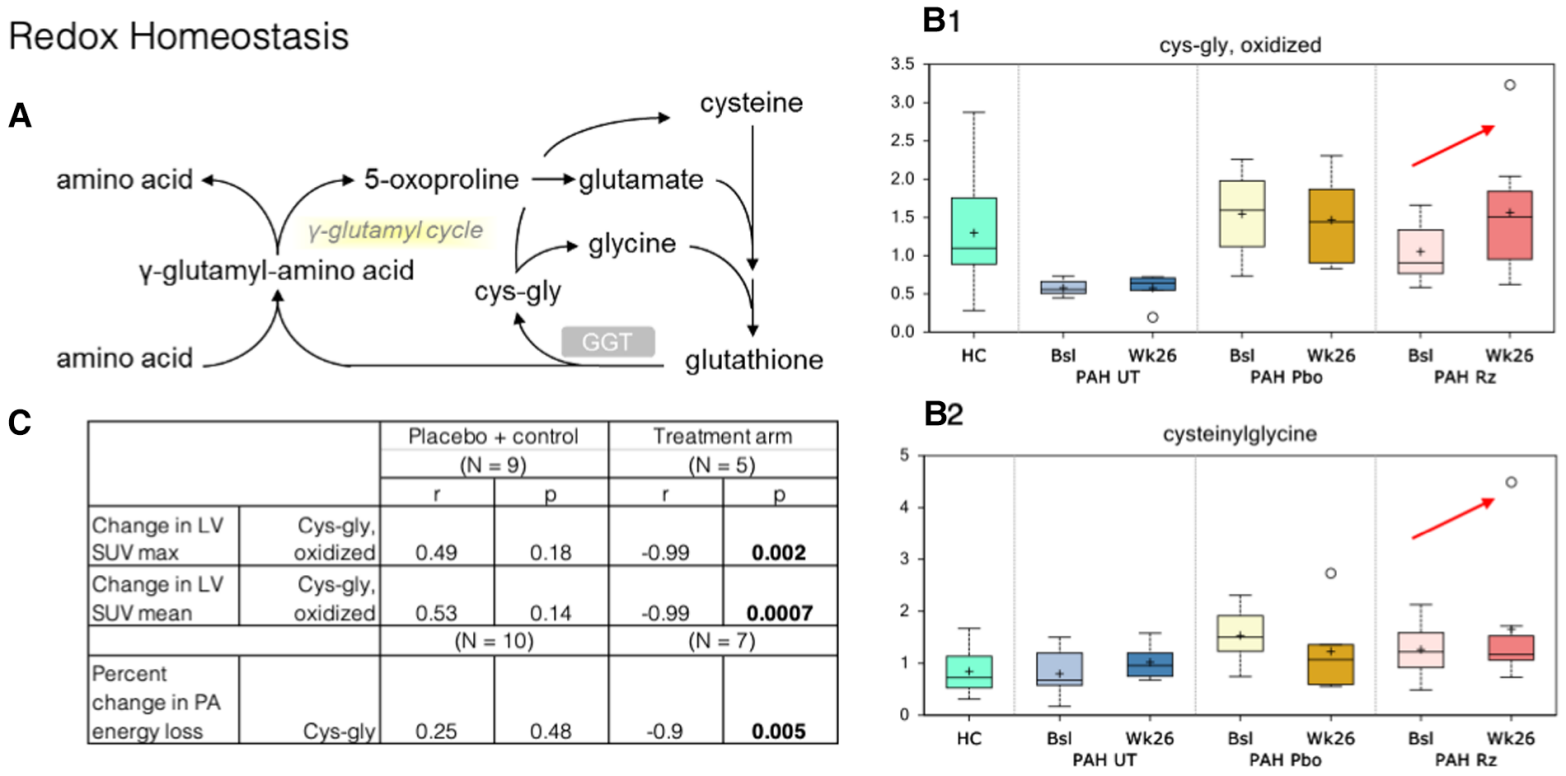
Correlation of PET SUV uptake and fluid dynamic changes in the LV with redox homeostasis metabolism. (**A**) Redox homeostasis pathway. GGT, Gamma-Glutamyl Transferase. (**B**) B1–B2. Cysteinylglycine and oxidized cys-gly were higher at week 26 samples compared to that observed at baseline (red arrows). HC, healthy control. Bsl, baseline; wk26, week 26; PAH, pulmonary arterial hypertension; Pbo, placebo; Rz, ranolazine. (**C**) Correlations observed in ranolazine's effect on various metabolites and changes in myocardial glucose consumption and fluid dynamics. LV, left ventricle; SUV, standardized uptake values; cys-gly, L-cysteine-L-glycine.

**Figure 4:**
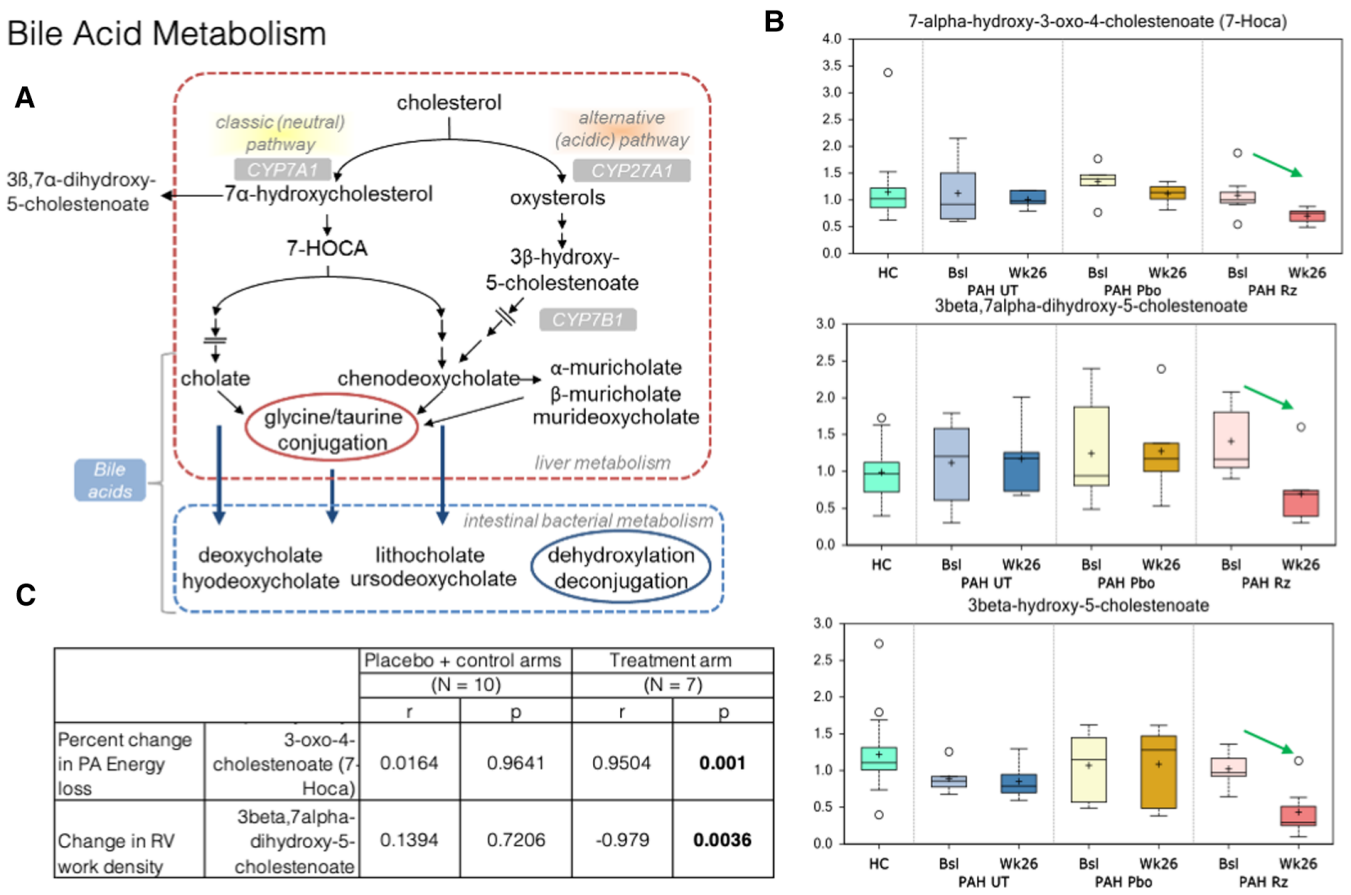
Strong association is present in decrease in 7-alpha-hydroxy-3-oxo-4-cholestenoate (7-HOCA) and decrease in PA energy loss in ranolazine treated patients. (**A**) Bile acid metabolism pathways. (**B**) Significant decreases in 7-alpha-hydroxy-3-oxo-4-cholestenoate (7-HOCA), 3beta-hydroxy-5-cholestenoate, and 3beta,7alpha-dihydroxy-5-cholestenoate between the week 26 and baseline samples collected from the ranolazine-treated subjects. HC: healthy control. Bsl, baseline; wk26, week 26; PAH, pulmonary arterial hypertension; Pbo, placebo; Rz, ranolazine. (**C**) Correlations observed in ranolazine's effect on 7-HOCA and fluid dynamic measurements. There was no association in placebo and controls subjects, but the association was strong decrease of in 7-HOCA and decrease in PA energy loss (*r* = 0.95; *p* = 0.001) in ranolazine-treated subjects. Additionally, there was a significant and highly negative correlation between the changes in RV work density and 3beta,7alpha-dihydroxy-5-cholestenoate level (*r* = −0.98, *p* = 0.004) in the ranolazine treatment group.

**Figure 5:**
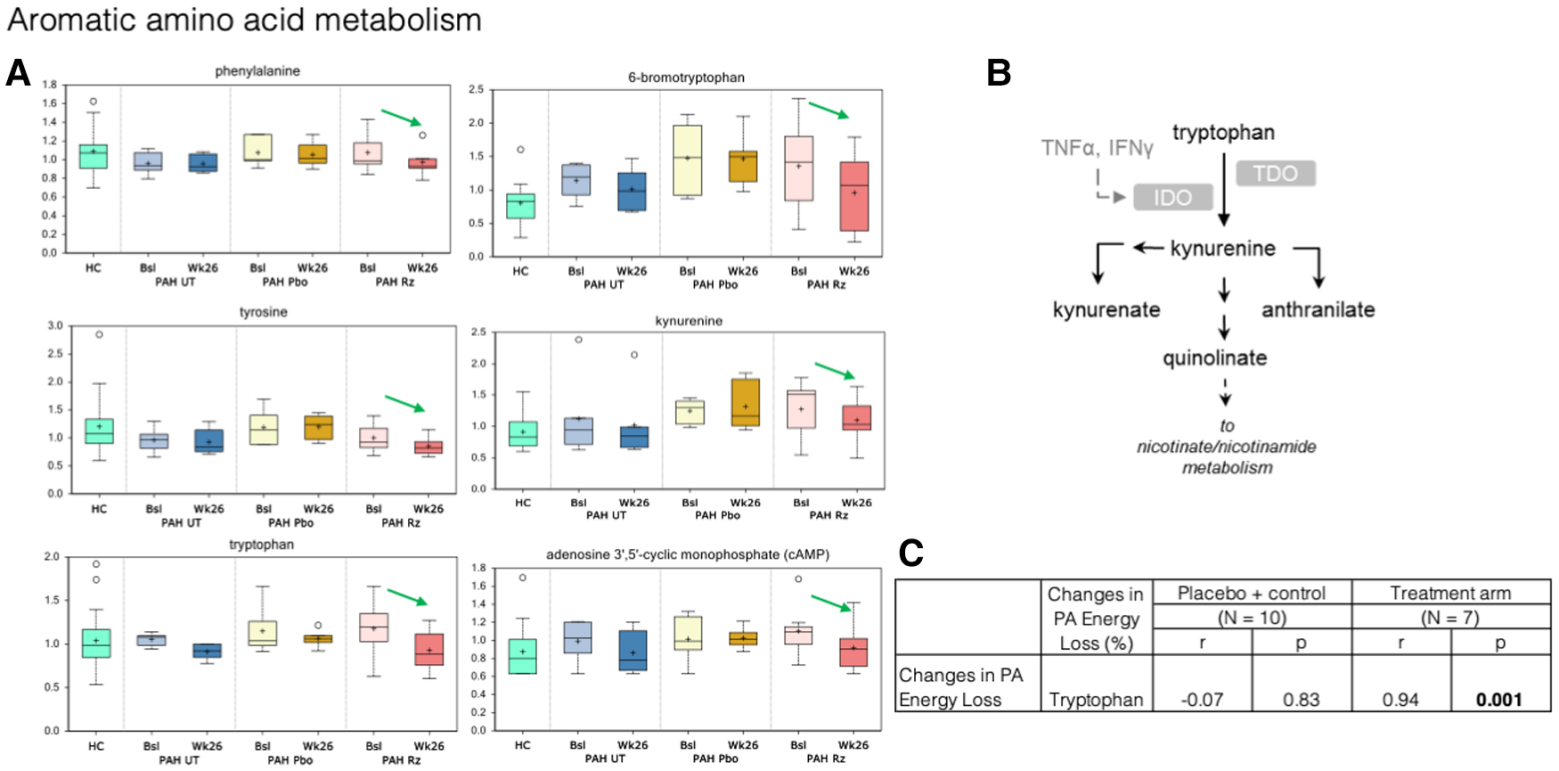
Treatment with ranolazine was found to be associated with decreases in circulating levels of the certain aromatic amino acids. (**A**) Treatment with ranolazine was found to be associated with decreases in circulating levels of several compounds in the tryptophan pathway (green arrows). In contrast, no statistically significant changes were observed for these metabolites in the untreated and placebo groups. (**B**) Tryptophan metabolic pathway. (**C**) Decrease in PA energy loss was significantly correlated with decreased levels of tryptophan in the ranolazine group (0.94; *p* = 0.001).

Metabolomic changes observed in plasma obtained from PAH patients treated with ranolazine involved several metabolic pathways: aromatic amino acid metabolism, redox homeostasis, bile acid metabolism, lipid metabolism, and modified amino acids, as summarized in Figures 3–5.

In redox homeostasis, we noted changes in glutathione-derived metabolites within the ranolazine-exposed group: cysteinylglycine, cysteinylglycine disulfide, and oxidized cysteinylglycine were higher in the week-26 samples compared to that observed at baseline. Cysteinylglycine and oxidized cysteinylglycine were corelated with higher baseline RV/LV mean FDG uptake (*r* = 0.67, 0.54; *p* = 0.002, 0.016). Additionally, a higher level of oxidized cysteinylglycine has a strong negative correlation (*r* = −0.99, *p* = 0.002) with decreased SUV max in the LV but not the RV, and a higher level of cysteinylglycine has a strong negative correlation with decreased energy loss in the PA (*r* = −0.9, *p* = 0.005).

In bile acid metabolism, we detected significant decreases in 7-alpha-hydroxy-3-oxo-4-cholestenoate (7-HOCA), 3beta-hydroxy-5-cholestenoate, and 3beta,7alpha-dihydroxy-5-cholestenoate between the week 26 and baseline samples collected from the ranolazine-treated subjects (*p* = 0.02, 0.0001, 0.0004). The latter two compounds had q values, or the false positive rates <10%. Furthermore, there is a significant and highly positive correlation between the changes in PA energy loss and 7-HOCA level (*r* = 0.95, *p* = 0.001), and a significant and highly negative correlation between the changes in RV work density and 3beta,7alpha-dihydroxy-5-cholestenoate level (*r* = −0.98, *p* = 0.004) in the ranolazine treatment group.

In aromatic amino acid metabolism, treatment with ranolazine was found to be associated with decreases in circulating levels of phenylalanine, tyrosine, tryptophan, kynurenate, kynurenine, and cAMP; additionally, there was also significant decrease in circulating level of 6-bromotryptophan, a bi-product in the tryptophan metabolism in the ranolazine group (Figure 5). In contrast, no statistically significant changes were observed for these metabolites in the untreated and placebo groups. Decrease in PA energy loss was significantly correlated with decreased levels tryptophan, in the ranolazine group (*r* = 0.94, *p* = 0.001). A higher level in 6-bromotryptophan is associated with a higher LV SUV max in the control group (*r* = 0.83, *p* = 0.006).

## Discussion

For most PAH patients, long-term survival and quality of life depends on the ability, largely through pharmacological treatments, to preserve RV function. In this study, we integrated multi-modality imaging techniques to investigate changes in fluid dynamics, targeted cardiac metabolism, and systemic metabolomics pre and post treatment in patients with PAH. The number of subjects in each arm of this study is small; nonetheless, the results provide hypothesis generating insights into the mechanism in which early fluid dynamic and metabolic changes relate to biochemical changes in individuals following ranolazine treatment and its potential implications in downstream cardiac remodeling.

### Myocardial metabolism in heart failure

Metabolic derangement in left heart failure has been of major interest over the last two decades. Animal models and clinical studies suggested changes in substrate utilization, oxidative phosphorylation, and high-energy phosphate metabolism (16–18). A healthy heart largely relies on oxidative phosphorylation from fatty acid oxidation. As heart failure progresses, there are decreases in fatty acid utilization and oxygen consumption, as well as an initial upregulation of glycogenolysis to maintain energy production but a subsequent decrease as the disease progresses. This provides the basis for PET to detect metabolic abnormalities in right heart failure using glucose analog ^18^F-FDG.

Growing evidence suggests that RV cardiomyocyte metabolic state plays an important role in determining the RV adaptive/maladaptive response (19). Many human studies and animal models have demonstrated that metabolic pathways are reprogrammed with vascular remodeling and molecular pathogenesis of PAH (20, 21). The RVs in PAH patients also exhibit significant metabolic shifts away from glucose oxidation to support hypertrophy and energy metabolism (22, 23), though this adaptation is not necessarily protective. The inhibition of pyruvate dehydrogenase kinase (PDK), which increases pyruvate dehydrogenase (PDH) activity and upregulates glucose oxidation, has been shown to partially restore RV function in animal models (24).

Ranolazine has been shown to alter cellular metabolism in animal models and small-scale human studies. In a mouse model for nonalcoholic fatty liver disease, ranolazine significantly increased glucose oxidation by upregulating PDH activity (25). Additionally, in *ex vivo* studies, ranolazine treatment in an isolated working heart model led to increased mitochondrial glucose oxidation and improvement RV function (26).

Our study showed that ranolazine had demonstrated a trend toward decreased RV/LV mean FDG uptake ratio over time. Previously studies have shown significant correlation between RV/LV FDG uptake ratio and pulmonary vascular resistance, PA pressure, RVEF, and PAH prognosis (27–30). Moreover, higher levels of RV FDG uptake are associated with more rapid clinical worsening of PH (31, 32).

### Redox homeostasis

PAH is characterized by arterial obstruction secondary to excessive proliferation and impaired apoptosis of pulmonary artery smooth muscle cells, endothelial dysfunction, inflammation, and excessive vasoconstriction (33). Reactive oxygen species play important roles in fibroblast activation and extracellular matrix deposition. Oxidative stress is associated with RV failure secondary to PAH, a more accentuated oxidative response to pressure overload compared to that of the LV (34–37). Animal studies have shown in SOD3 knot-out mice that are deficient in extracellular superoxide dismutase, develop significantly worse PAH (higher RV pressure and worse RV hypertrophy) under conditions of oxidative stress (38).

*γ*-Glutamyltransferase (GGT) is a biomarker that is elevated during oxidative stress and systemic inflammation, and it was previously shown to have some prognostic value in PAH (39). No changes in gamma-glutamyl amino acids were noted in our study, but the changes in cysteinylglycine disulfides (reduced) and oxidized species could be interpreted as an increase in GGT activity, which transfers the gamma-glutamyl moiety from glutathione to acceptor amino acids (Figure 3). However, it is possible that these increases originate from greater glutathione availability in subjects treated with ranolazine and an increased antioxidant defense capacity, especially that the higher level of cysteinylglycine was strongly associated with decreased energy loss in the PA seen in our study.

### Bile acid metabolism

Bile acid synthesis occurs through two pathways: the classic (neutral) pathway and the alternative (acidic) pathway. The classic pathways occur in the liver and accounts for about 90% of bile acid synthesis, while the alternative pathway is primarily extrahepatic, in vascular endothelium and macrophages (40, 41). Its presence in the lungs in PAH patients were previously thought to be partial reflux until a recent study identified elevated expressions of cytochrome P450 B1 (CYP7B1) in PAH lungs, suggesting that PAH lung tissue may have the capacity for *de novo* synthesis of bile acids, through the acid pathway of bile acid metabolism (42).

In our study, there is a significant and highly positive correlation between the changes in PA energy loss and 7-HOCA level; decreased energy loss in the form of viscous dissipation is almost perfectly linearly correlated with lower level of 7-HOCA in plasma in the ranolazine treatment arm. Blood is a non-Newtonian fluid, and its viscosity depends on the local stress condition (43). Under normal physiological states, intraventricular flow is aligned along the base-apex direction, consistent with the inflow-outflow path. This dynamic flow pattern is altered in pathological conditions in response to changes in hemodynamics. Energy loss in the PA in PAH patients arises from the shear stresses that transform mechanical energy into thermal energy, secondary to the intrinsic viscosity of blood. The amount of energy loss depends on flow pattern and velocity, which are sensitive to RV afterload conditions.

Elevated levels of 7-HOCA were previously observed in patients with porto-pulmonary hypertension and hepatopulmonary syndrome (44). As mentioned earlier, extrahepatic bile acid synthesis occurs in the vascular endothelium. The exact mechanism behind the activation of pulmonary bile acid synthesis is unknown, but it may be a consequence of endothelial dysfunction, which then act as steroid hormones and serve as substrates for nuclear membrane receptors and/or g-protein copied receptor signaling gene transcriptions for major enzymes in bile acid synthesis (such as CYP7A1 and CYP8B1) and other key proteins in cell cycle regulation—processes that have been shown in cancer metastases (45, 46). Buermans et al. showed in a monocrotaline-induced pulmonary hypertension rat model that early in the disease process, ventricles destined to progress to failure showed activation of pro-apoptotic pathways via the lack of MAPK phosphatase-1 upregulation in the p38-MAPK pathway—early differentiation of the compensated vs. decompensated phenotypes (47). Additionally, 7-HOCA is in the center stage of the bile acid synthetic pathway. It is also a microbiota derived metabolite implicated in the insulin and glucose metabolism (48), which might be important as the change of glucose metabolism (decreased RV uptake as shown with FDG) might lead to better RV kinetic energy utilization and lower PA energy loss.

Flow changes precede structural changes, but the precise mechanistic linkage is not yet known. Here, we suggest evidence that ranolazine alter key reactions in bile acid metabolism that is reflected in the fluid dynamic profile in the PA.

### Aromatic amino acid metabolism

Higher plasma levels of aromatic amino acids have been linked to increased risk of cardiovascular event and the development of insulin resistance (49, 50). Our results suggest that the beneficial effects of ranolazine can be, at least in part, manifested through declines in phenylalanine, tyrosine, and tryptophan metabolism. Additionally, we detected a subtle decrease in kynurenine level in the ranolazine group, which was recently reported as a marker of PH and was shown to induce an increase in adenosine 3′,5′-cyclic monophosphate (cAMP) levels in human pulmonary arterial smooth muscle cells (51). In fact, we detected a significant decrease in cAMP levels in the ranolazine cohort over the course of the treatment.

Nicotinamide adenine dinucleotide (NAD) is gaining increasing interests from the heart failure community as a potential therapeutic target (52), but mechanisms leading to altered NAD levels in heart failure is not fully understood. Tryptophan is a direct precursor for *de novo* NAD production. We showed that the decrease in PA energy loss was significantly correlated with decreased levels of tryptophan. As discussed earlier, the PA energy profile reflects the flow pattern in the RVOT and the PA. These fluid dynamic changes lead to subtle changes in local mechanical stress conditions, which may subsequently lead to mechanical sensor induced downstream signaling pathways that utilize increased levels tryptophan.

Taken together, these results indicate that ranolazine administration is associated with declines in circulating levels of kynurenine and cAMP, which may be secondary to beneficial effects of the drug on tryptophan metabolism and kynurenine-dependent signaling.

## Limitations

The study population is very small, and human-to-human variability may limit the number of statistically significant observations in the metabolomic analysis.

## Conclusion

In this study, we showed that ranolazine alters metabolism in patients with precapillary pulmonary hypertension using a multimodality approach with CMR and PET imaging and metabolic profiling. Despite the small study population, our study suggests that the beneficial effects of ranolazine: 1. Decreasing glucose uptake in the RV; 2. Regulating redox homeostasis towards more robust defense mechanism against oxidative stress; 3. Favorable effect on the pulmonary blood flow associated with key changes in bile acid and aromatic amino acid metabolism. Larger patient studies are needed to validate the beneficial effects of ranolazine, and animal models for biochemical pathways are needed to investigate the details of mechanisms suggested in this work.

## Data Availability

The original contributions presented in the study are included in the article, further inquiries can be directed to the corresponding author.
